# Development and validation of the Essen Melanoma Quality of Life Inventory (EMQoLI)

**DOI:** 10.1111/ddg.15998

**Published:** 2026-04-20

**Authors:** Venja Musche, Theresa Schadendorf, Adam Schweda, Georg Lodde, Jessica Neumann, Lisa Zimmer, Svea Hüning, Dirk Schadendorf, Eva‐Maria Skoda, Elisabeth Livingstone, Martin Teufel

**Affiliations:** ^1^ Clinic for Psychosomatic Medicine and Psychotherapy LVR‐University Hospital Essen University of Duisburg‐Essen Essen Germany; ^2^ Center for Translational Neuro‐ and Behavioral Sciences (C‐TNBS) University of Duisburg‐Essen Essen Germany; ^3^ The German Cancer Consortium Campus Essen Germany & National Center for Tumor Diseases (NCT‐West) Campus Essen & Research Alliance Ruhr Research Center One Health Campus Essen Essen Germany; ^4^ Department of Dermatology University Hospital Essen Essen Germany; ^5^ Department of Dermatology Hospital of Dortmund Dortmund Germany

**Keywords:** Cancer, Melanoma, Psychometrics, Quality of Life, Surveys and Questionnaires

## Abstract

**Background and Objectives:**

New treatments for melanoma, such as targeted therapy and immunotherapy, present unique adverse events compared to traditional treatments like surgery and chemotherapy. Existing health‐related quality of life (HRQoL) measures often fail to address these new treatment effects and focus primarily on chemotherapy and surgery. This study aimed to develop and validate a new HRQoL instrument for melanoma patients across all tumor stages, encompassing a broader range of adverse events and melanoma‐specific aspects.

**Patients and Methods:**

The Essen Melanoma Quality of Life Inventory (EMQoLI) was tested with 387 German‐speaking malignant melanoma patients across all tumor stages.

**Results:**

Exploratory factor analysis revealed a three‐factor structure. Eight items were removed due to low or cross‐loading, leaving a final version of 25 items with satisfactory reliability and internal consistency. Correlation analyses indicated good convergent and discriminant validity.

**Conclusions:**

The EMQoLI is a 25‐item German self‐report tool based on a 5‐point Likert scale, designed to assess HRQoL in melanoma patients across all tumor stages. It offers healthcare professionals an efficient tool to identify patients needing psychosocial support and can serve as a standardized measurement instrument in clinical settings.

## INTRODUCTION

Melanoma accounts for around 57,000 attributable deaths in 2020.^1^ About 325,000 new melanoma cases were diagnosed in 2020, and demographic forecasts suggests that the incidence will increase by over 50 % by 2040.^1^ In recent decades, treatment options have evolved from surgical removal, radiation therapy, and conventional chemotherapy to targeted therapies and immunotherapies.^2^, ^3^, ^4^ Although these evolving therapies control symptoms, delay disease progression, and ultimately improve survival rates of late‐stage melanoma patients, they are also associated with different toxicity profiles and serious treatment‐related adverse events (TRAE), such as colitis, hepatitis, hypophysitis, endocrinopathies, cardiac and skin toxicity, or retinopathy.^2^, ^5^, ^6^, ^7^, ^8^ Additionally, many patients now receive these treatments in an adjuvant setting, where the risk of recurrence and melanoma‐related death is lower, meaning that the consequences of the disease and its treatment must be considered differently.

After surgical treatment, melanoma patients remain at an increased risk of tumor recurrence, potential metastasis, or disease progression for many years, necessitating ongoing follow‐up.^2^, ^9^ The impact of melanoma varies depending on tumor stage; for example, early‐stage thin melanomas may require only surgical excision and follow‐up consisting of regular skin examinations and lymph node palpation.^2^, ^10^, ^11^ In contrast, patients with higher‐stage melanoma with a significant risk of recurrence undergo regular radiological imaging and must remain vigilant about the possibility of recurrence.^2^, ^9^, ^10^, ^11^ Advanced‐stage patients can also experience symptoms of metastases, and those patients who receive systematic treatment may suffer from adverse treatment effects.^2^ Despite advances in treatment, therefore, many melanoma patients continue to face additional burdens, including also financial and social worries. Melanoma must therefore be regarded as a chronic, life‐threatening disease that substantially impacts patients’ *health‐related quality of life (HRQoL)*.^7^, ^12^, ^13^

HRQoL is multidimensional. It includes physical and social functioning as components of health, as well as psychological and behavioral components of well‐being and overall quality of life affected by illness or treatment.^14^, ^15^, ^16^ As patient‐reported outcome measures, HRQoL assessments have gained importance in health and cancer research.^16^, ^17^ Measures of HRQoL provide complementary, subjective information to more traditional, medical parameters in areas where TRAE and long‐term survival can significantly affect patients’ lives and functioning.^17^ Therefore, assessing HRQoL in melanoma patients should be integral to routine diagnosis, follow‐up, and clinical trials in melanoma patients, providing valuable information on the subjective effects of the disease and treatment to support medical decision‐making.^17^

Generally, numerous measurement tools for HRQoL exist.^17^ These are mainly generic, rather than being specific to cancer patients or melanoma patients. Melanoma‐specific issues such as the tumor stage, the corresponding treatment type, TRAE, or sun‐avoidant behaviors are not considered in generic HRQoL measures. Therefore, existing HRQoL measures cannot adequately reflect melanoma‐specific HRQoL.^5^, ^18^, ^19^, ^20^ Studies examining melanoma patients often use cancer‐specific HRQoL questionnaires such as the *European Organisation for Research and Treatment of Cancer Quality of Life Questionnaire* (EORTC‐QLQ‐C30).^19^, ^20^, ^21^, ^22^ However, the assessment of melanoma‐specific HRQoL could additionally be influenced by factors beyond the generic measures of disease progression and treatment success assessed by the EORTC‐QLQ‐C30, such as the public's lack of understanding and recognition of melanoma.^5^, ^18^, ^19^, ^20^ Hence, even cancer‐specific HRQoL questionnaires may not be sensitive enough to detect melanoma‐specific effects.

Few melanoma‐specific HRQoL instruments are available. Based on the EORTC‐QLQ‐C30, the *EORTC Melanoma Module* (EORTC‐QLQ‐MEL38)^19^ was developed, followed by a refinement in 2019, the *Melanoma Concerns Questionnaire* (MCQ‐28).^7^ However, the validation of these measurement tools has been criticized.^7^, ^19^ The most frequently used melanoma‐specific HRQoL measurement instrument is the *Functional Assessment of Cancer Therapy‐Melanoma subscale* (FACT‐M).^23^, ^24^ Refinement of both the structure and response format of the FACT‐M has been repeatedly recommended.^7^, ^25^ Moreover, research indicates that lower HRQoL is related to present mental health issues and unmet care requirements. These are not examined in the FACT‐M, suggesting it to be less comprehensive.^5^, ^26^ Additionally, the FACT‐M was originally designed for clinical trials examining melanoma patients with higher tumor stages and higher mortality rates, which thus receive additional surgical or systemic therapy.^27^ Hence, another concern is that current instruments assessing melanoma‐specific HRQoL primarily focus on the TRAE of chemotherapy and surgery and may not fully capture TRAE related to newer, melanoma‐specific therapies such as immunotherapy or targeted therapy.^5^, ^28^

In summary, existing HRQoL instruments may not adequately address aspects specific to melanoma patients, such as the direct impact of melanoma symptoms, tumor stages, the treatment‐related burden of therapies, or the psychological burden of melanoma diagnosis.^2^, ^5^, ^26^, ^28^, ^29^, ^30^ To address these issues, a new melanoma‐specific HRQoL questionnaire, namely the *Essen Melanoma Quality of Life Inventory* (EMQoLI), was developed. The aim of this study is to assess the structure and psychometric properties of the newly developed measurement instrument in a large sample of melanoma patients with different tumor stages.

## METHODS

### Participants and study design

Participants were recruited from December 2019 to January 2023 in the Departments of Dermatology at the University Hospital Essen and the Hospital of Dortmund, and in online support groups for melanoma patients. The study was based on a comprehensive online survey, which included multiple standardized questionnaires as well as self‐constructed items, and was conducted via the Unipark platform (Tivian XI GmbH, Berlin). The survey constituted a central element of the study, extending beyond the scope of the Essen Melanoma Quality of Life Inventory (EMQoL, which is the focus of this article. All participants provided electronic, informed consent before voluntarily participating in the anonymous self‐report study. Inclusion criteria were *(1)* a verified melanoma diagnosis, *(2)* 18+ years old, and *(3)* sufficient German language skills. The final sample consisted of 387 participants. The mean duration of the entire survey was 26 minutes and 46 seconds (SD, 11:59). The cross‐sectional evaluation study was approved by the Ethics Committee of the Medical Faculty of the University of Duisburg‐Essen (19‐8862‐BO).

### Development of the EMQoLI

Current literature on melanoma patients' HRQoL, related measurement tools, and input from a multi‐professional expert panel including nurses and physicians working with melanoma patients were considered for item creation.^20^, ^29^, ^30^, ^31^ All items were administered in German. Prior to the main study, a pilot study (n = 59 melanoma patients across different tumor stages) was conducted to assess item quality, acceptability, scope, and questionnaire length (see supplemental online material). Based on the results, the length of the questionnaire was adjusted, and some items were rephrased to ensure better comprehensibility. The final items were selected by consensus after passing through several expert feedback loops. The expert team consisted of psychologists with a background in test construction, physicians and nurses specialized in dermatooncology. The preliminary version of the EMQoLI consisted of 33 items, answered on a 5‐point Likert‐scale from 0 = “strongly disagree” to 4 = “strongly agree” with higher scores indicating lower HRQoL. Seven items were inverted to reduce response bias and improve data quality. The questionnaire instructions specified that items should be answered in relation to the previous two weeks. Completion of the EMQoLI takes on average around five minutes. The final German version of the EMQoLI, along with a preliminary English version, is available in the supplemental online material.

### Study variables

Sociodemographic and medical information was assessed for sample description. The following assessment instruments were applied to test the convergent and discriminant validity of the EMQoLI, i.e. its overlap with related measures and divergence with non‐related measured.

*European Organization for Research and Treatment of Cancer Core Quality of Life Questionnaire (EORTC‐QLQ‐C30)*.^20^ The EORTC QLQ‐C30 is a validated multidimensional instrument for assessing quality of life in oncological patients, comprising functional subscales, symptom subscales, a health status/global quality of life scale, and single items. We expect the newly developed EMQoLI and the EORTC QLQ‐C30 to show a significant positive relationship.

*Hospital Anxiety and Depression Scale (HADS)*.^31^ The HADS is a validated screening instrument where anxiety and depression symptoms are assessed with seven items each. We assume that anxiety and depression would go along with an impaired melanoma‐related quality of life, manifesting in a positive association with the EMQoLI.

*Generalized Self‐Efficacy Scale (GSE)*.^32^ The German version of the GSE, consisting of ten items rated on a 4‐point Likert scale, is a validated self‐report measure of self‐efficacy and optimistic self‐beliefs related to coping with a variety of difficult life demands as well as coping with all types of stressful life events. Here, we expect a high negative relationship with the EMQoLI.

*Barratt Impulsiveness Scale – Short Version (BIS‐15)*.^33^ The BIS‐15 consists of 15 items, answered on a 4‐point Likert scale, and measures three factors: non‐planning impulsivity, motor impulsivity and attention‐based impulsivity. Here, we assume a non‐significant or low correlation between impulsiveness and the EMQoLI.

### Data analysis

The data analysis was conducted using R version 4.4.1.^34^ Since this is the first validation study of the EMQoLI, an exploratory factorial approach was used for test construction and extraction of respective subscales. Here, the *n_factors* command from the R package *parameters*,^35^ was used, which automates the application of many procedures to extract the optimal number of factors (e.g., parallel analysis, Velicer's MAP). Subsequently, *exploratory factorial analyses* (EFA) were performed using standard procedures from the *psych* R package,^36^ applying a varimax‐rotation based on polychoric correlation matrices. Item selection was performed based on factor loadings, for which a cutoff of 0.4 was defined, as well as the items’ communalities (cutoff: < 0.25). For the final instrument, MacDonald's omega as a measure of reliability was reported. Spearman‐correlation coefficients illustrate the measure's convergent or discriminant validity. Mann‐Whitney‐U‐Tests and Kruskal‐Wallis‐Tests were computed to assess differences of test scores in disease‐specific and sociodemographic categories (e.g., tumor stage, sex). The sample size of 387 participants was in accordance with recommendations for validation studies.^37^

## RESULTS

### Sample characteristics

A total of 429 patients participated, of which 42 were excluded due to an unclear melanoma diagnosis. The remaining 387 participants of this study had a mean age of M = 54.12 years (SD = 13.39). Melanoma patients of stages I‐IV participated, with 28.7 % having locoregional metastasis (stage III) and 33.6 % having distant metastatic disease (stage IV). Within this sample, 57.9 % were currently tumor‐free. See Table 1 for further socio‐demographic and medical characteristics of the study sample.

**TABLE 1:** Sociodemographic and medical characteristics at the time of the survey.

	M	SD
*Age* ^a^	54.12	13.39
	n^b^	%^b^
*Sex* ^c^	212	54.80
*Marital status*		
Single	57	14.70
Married	243	62.80
In a relationship	37	9.60
Divorced/separated	34	8.80
Widowed	16	4.10
*Children* ^d^	267	69.00
*Educational level*		
University education	80	20.70
Higher education entrance qualification	121	31.30
Intermediate secondary education	095	24.50
Lower secondary education	085	22.00
None	6	1.60
*Employment*		
Partial employment	60	15.50
Full employment	134	34.60
In apprenticeship	3	0.80
Retired	116	30.00
Housewife/‐husband	4	1.00
Not employed	13	3.40
Sick leave	38	9.80
Other	19	4.90
*Tumor stage*		
I	85	22.00
II	61	15.80
III	111	28.70
IV	130	33.60
*Treatment situation* ^e^		
Tumor‐free	224	57.90
Non‐resectable disease/metastastic disease detectable	62	16.00
Could not be assessed	101	26.10
*Current type of treatment* ^f^		
None/ Follow‐up care	133	30.80
Surgery	73	16.90
Immunotherapy	173	40.00
Targeted therapy	31	7.20
Radiation therapy	7	1.60
Other therapies	15	3.50

*Abbr*.: M, mean; SD, standard deviation

^a^ *Note*: mean and standard deviation reported

^b^ subsequent columns refer to the sample (n) and demonstrate the number of test participants, or the percentage of the sample, who possess specific characteristics

^c^ regarding female sex

^d^ regarding people with children

^e^ Patients had to self‐report their current treatment situation using the terms curative and palliative. Table 1 shows the corresponding medical terminology used and provided by the treating dermatologists

^f^ more than one treatment type was possible.

### Test construction

First, simple item‐wise descriptive statistics are reported. This is followed by an exploratory factorial and reliability assessment. Associations were explored to gauge convergent and divergent validity using measures of similar and different constructs.

### Basic descriptive and psychometric analysis

Summary statistics of the final version of the EMQoLI, including all 33 items, were computed. Most of the items display a non‐negligible right‐skewed distribution, which is not uncommon in psychological symptom diagnostics,^38^ and quality of life measurement.^39^ This indicates that the EMQoLI captures an impairment‐related variable rather than a general trait. Moreover, the comparably low variance of symptom‐related items – particularly those referring to acute nausea, regurgitation, diarrhea, and fever – indicates a low prevalence of these symptoms in the present sample. Therefore, the present study relied on polychoric correlations for the EFA.^40^

### Item Selection and factorial validation

As a first step to validation, we extracted the items that are most indicative of melanoma‐related quality of life (and the respective subdomains). Hence, to initially assess the optimal number of items, as well as factorial stability, we conducted a first EFA based on the number of factors proposed by the highest consensus of various retention procedures implemented in the *n_factors* command from the *parameters* package.^35^ All items show Kaiser‐Meyer‐Olkin values of above 0.6, with most of the items ranging above 0.9, suggesting high suitability for factorial analyses, in general. Here, four out of 19 methods (21.05 %) suggest an optimal factor number of three. Eight items needed to be excluded due to cross‐loadings and low communalities (see online supplementary Table S1), indicating ambiguousness or relative redundancy. Due to the non‐specificity but high relevance of fatigue symptoms, item number three (“I suffered from tiredness”) was retained within the factor with the highest loading despite the cross‐loading. Item number two (“I have swellings in the area of the postoperative scar of the melanoma and/or the metastases”) was retained despite a lower communality of 0.235 due to its relevance for an individual's overall level of functioning and, thus, quality of life.

The remaining 25 items were again evaluated as to the optimal number of factors. Here, seven out of 19 (36.8 %) procedures that were used for determining the optimal number of factors support a three‐factor solution. A subsequent factorial analysis – again using a varimax rotation – shows that all items have loadings above 0.4 and communalities above 0.25 (see online supplementary Table S2), suggesting that each item can be unambiguously assigned to one subscale and no items appear redundant. Only item three (“I suffer from tiredness”) exhibits significant cross‐loadings, which we deem acceptable due to the relative non‐specificity of tiredness as a symptom. The proportion of explained variance due to the factorial structure is 49 %.

The final EMQoLI consists of 25 items, being scored on a five‐point Likert‐scale. Six items are inverted. Based on the respective items included, the three EMQoLI subscales were interpreted as measuring *Symptoms, Burden* and *Resources*. Higher EMQoLI scores indicate higher impairment of HRQoL.

### Reliability analyses

Reliability indices also indicate a satisfying level of reliability (see supplemental online material). All three subscales show modest to moderate correlations with other subscales (Burden x Symptoms rho = 0.45, p < 0.001; Burden x Resources rho = 0.26, p < 0.001; Resources x Symptoms rho = 0.22, p < 0.001).

### Validity of the EMQoLI‐scale

#### Associations with sociodemographic and health‐related characteristics

Female participants show elevated scores on each of the subscales of the EMQoLI, as well as the total score (all p < 0.05), whereas the difference on the scale Burden is most pronounced (Mann‐Whitney U test: W = 24880, p < 0.001). Also, a Spearman correlation analysis assessing the relationship between participants’ age and EMQoLI‐scores shows that Burden decreases with the participants’ age (rho = ‐0.242, p < 0.001), an effect that possibly drives a significant correlation between age and the EMQoLI total score (rho = –0.219, p < 0.001). See the supplemental material for further details regarding the association with further sociodemographic and health‐related characteristics.

### Differences between tumor stages

**FIGURE 1:**
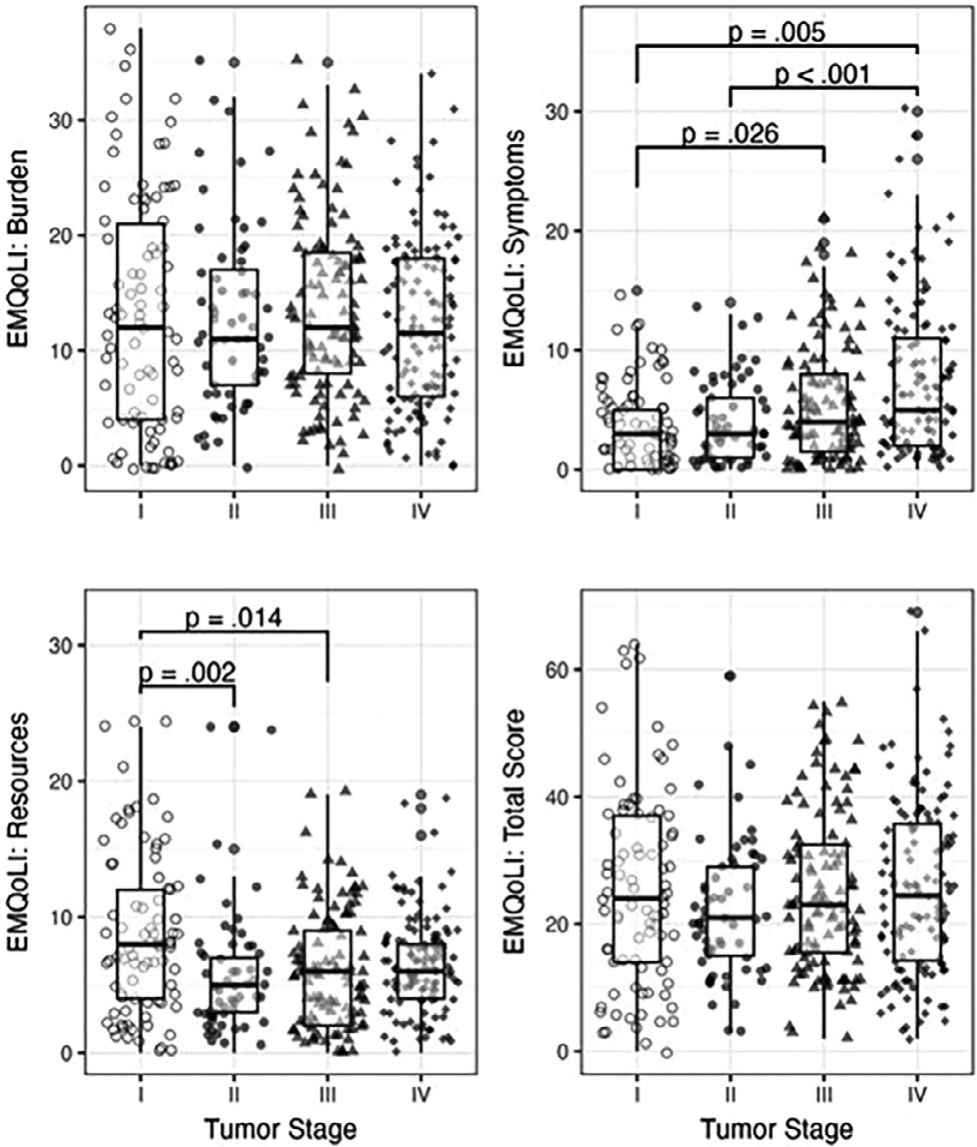
Differences in EMQoLI scores across the four tumor stages. Significant differences were observed in the amount and severity of symptoms present (upper right panel) as well as in perceived resources.

To assess whether the perceived HRQoL is associated with the severity of the disease, we compared the the EMQoLI scores among patients with tumor stages I to IV. Due to the distributions’ skew, Kruskal‐Wallis‐Tests were computed. No differences were found between patients with different tumor stages for neither the total EMQoLI score (χ^2^(3) = 2.512, p = 0.47), nor for the Burden subscale (χ^2^(3) = 1.761, p = 0.62). Yet, significant differences were found between tumor stages for the subscale Symptoms (χ^2^(3) = 22.978, p < 0.001). Subsequent Holm‐corrected post‐hoc Dunn's tests reveal significant differences between tumor stages I and III (z = 2.72, p = 0.026), tumor stages I and IV (z = 4.33, p < 0.001), as well as tumor stages II and IV (*z* = 3.32, *p* < 0.001). Significant differences can also be found for the *Resources* subscale (χ^2^(3) = 14.97, p = 0.002). Here, the respective post hoc tests reveal significant differences between tumor stages I and II (*z = *–3.57, *p* = 0.002), as well as I and III (*z* = –2.98, p* = *0.014). These group differences are displayed in Figure 1.

### Convergent and discriminant validity

Further, we used additional psychometric tests to explore how the EMQoLI relates to other measures that are either similar (such as other HRQoL measures; or “construct‐related”, such as levels of depression and anxiety), or unrelated to the construct (such as impulsivity).

### Generic measures of HRQoL

**FIGURE 2:**
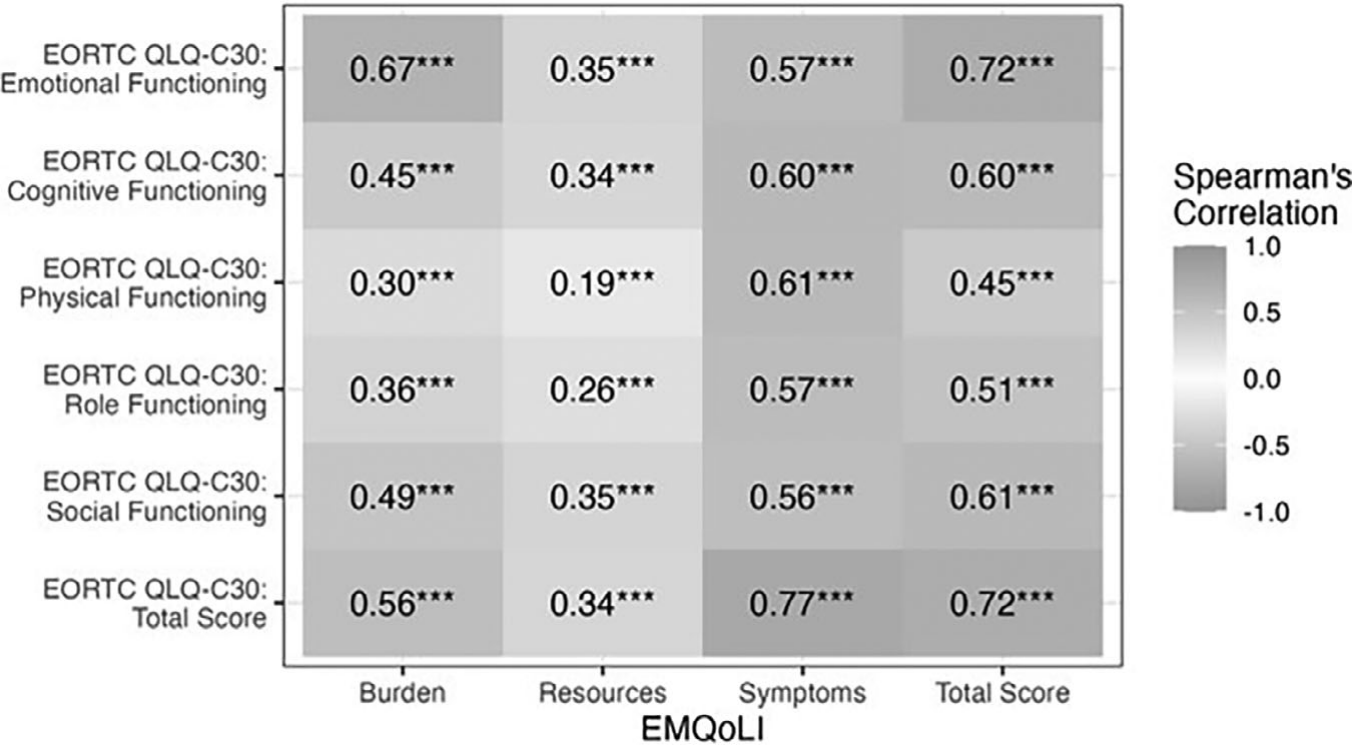
Holm‐corrected correlations between EMQoLI and the EORTC QLQ‐C30 scales.

Spearman‐correlation coefficients were calculated for each subscale and the total score of the EMQoLI and the EORTC QLQ‐C30. The results are presented in the correlation plot in Figure 2. All coefficients are statistically significant and suggest that high EMQoLI scores go along with high EORTC QLQ‐C30 scores. Note that the EMQoLI scale *Resources* shows the lowest overall correlations with the EORTC QLQ‐C30 scales.

### Indicators of mental health

**FIGURE 3:**
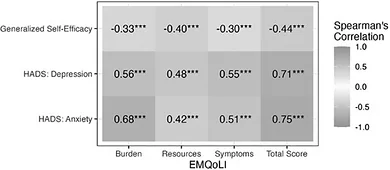
Holm‐corrected correlations between EMQoLI and mental health markers.

Spearman‐correlation coefficients were computed to explore the association between the EMQoLI (and its subscales), and levels of anxiety, depression, as well as general self‐efficacy – an important protective factor in the context of mental health. Correlations between the EMQoLI and self‐efficacy all turn out to be negative (Figure 3). The EMQoLI‐scores are positively associated with anxiety and depression.

**FIGURE 4:**
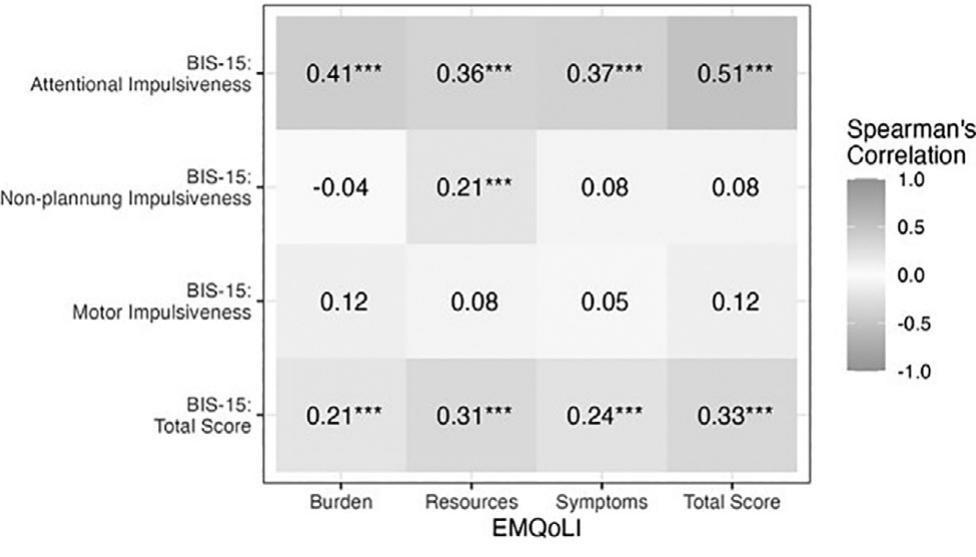
Holm‐corrected correlations between EMQoLI and BIS‐15.

To examine divergent validity, the correlations between the EMQOLI and impulsiveness, as measured by the BIS‐15 were analyzed (Figure 4). Significant associations were predominantly observed between the EMQoLI and attentional impulsiveness.

## DISCUSSION

The absence of somatic complaints after melanoma treatment does not automatically equate to good HRQoL. Melanoma therapy and assessments of HRQoL should encompass not only somatic factors such as pain, but also psychological aspects like fear of recurrence, as well as psychosocial elements including social support.^7^, ^12^, ^28^, ^41^ Therefore, the aim of the present study was to develop and validate an instrument to measure HRQoL in melanoma patients, regardless of tumor stage, that includes the TRAE of new treatments as well as psychological distress, psychosocial aspects, uncertainty and worry.

A large sample of German melanoma patients participated in the development and validation of the EMQoLI. An exploratory factorial analysis showed an overall good fit for a three‐factor model with 25 items grouped into three subscales: *Burden*, *Resources* and *Symptoms* (see online supplementary Table S2). The EMQoLI and its subscales showed satisfactory internal consistency in reliability analyses. Moreover, correlational analyses showed good convergent and sufficient discriminant validity. Significant correlations between the EMQoLI, EORTC QLQ‐C30, and HADS scales for anxiety and depression indicated convergent validity, as did a negative correlation with the German GSE, indicating that higher melanoma burden correlates with lower perceived self‐efficacy. Discriminant validity was demonstrated by low correlations with the BIS‐15 motor and non‐planning impulsivity subscales. Importantly, the BIS‐15 attentional impulsivity scale includes items that remotely measure symptoms of anxiety and agitation, which are similarly assessed on the HADS. Given this overlap, the positive correlation between the EMQoLI and the attentional impulsivity scale does not appear to indicate poor discriminant validity. Therefore, the attentional impulsivity scale of the BIS‐15 is not ideal for measuring discriminant validity. As this study is the primary evaluation of the EMQoLI, further psychometric research is recommended.

In this study, which included participants across all four tumor stages, female participants had significantly higher EMQoLI scores, indicating lower HRQoL. This effect was particularly pronounced on the *Burden* scale. This finding aligns with previous research describing gender differences in melanoma burden.^27^, ^42^ Furthermore, a significant, negative correlation was found between age and *Burden*, consistent with existing research.^43^ Higher tumor stages were associated with significantly greater physical distress on the *Symptoms* scale, reflecting findings that link tumor stage to somatic symptoms.^9^ On the *Resources* scale, tumor stage I patients reported feeling less supported by their personal environment and medical staff, and appeared less confident about the future compared to stage II and III patients. This finding is in line with previous research indicating that early‐stage patients may receive less psychosocial attention due to the comparatively favorable prognosis.^26^ However, although some group differences on the *Resources* scale were observed, these did not show a consistent or linear association with tumor stage. The overlap between groups, as illustrated by the wide distribution on the boxplots, suggests substantial individual variability.^44^ However, regardless of tumor stage, the EMQoLI identifies individuals with high physical and psychological quality of life burden and limited resources, demonstrating its applicability across all tumor stages.

Several limitations must be acknowledged when interpreting these findings. The number of participants by stage of melanoma is not uniform within the present sample (Table 1), with a higher proportion of stage III (28.70 %) and stage IV (33.60 %) patients compared to stage I (22.00 %) and stage II (15.80 %). In comparison, approximately 75 % of melanoma patients in Germany have stage I melanoma.^45^ The present sample also shows a lower mean age than the general melanoma population in Germany.^45^ Study participation was voluntary, and most participants were recruited through tertiary reference center specialized in the treatment of skin cancer, potentially introducing selection and self‐selection biases. Additionally, the study was conducted during the COVID‐19 pandemic, which extended the survey period to approximately three years. Although research indicates that the COVID‐19 pandemic did not significantly impact cancer patients’ distress or anxiety compared to healthy controls,^46^ the survey time and COVID‐19 pandemic are factors to consider. Furthermore, the present study relied on self‐reported data, which may limit objective verification of melanoma diagnosis, tumor stage, and treatment. However, participants from tertiary reference centers could confirm their diagnosis or treatment information by their local dermato‐oncologist if needed. Lastly, the questionnaire has only been validated in German and therefore cannot be used in other languages. An English validation of the questionnaire is in progress and will soon be available to English practitioners and patients.

In summary, melanoma therapy has progressed rapidly and with it the potential for TRAE. The literature and the present study show that there is a need for psychosocial support and that melanoma disease and therapy may be associated with psychological distress. Early identification of psychological and somatic distress and the need for psychosocial support can have a positive impact on the quality of care and, in the long term, on quality of life and recovery. As such an instrument was lacking, the newly developed EMQoLI is an instrument that covers these aspects in their entirety, thus improving the care of melanoma patients. The EMQoLI was developed to address the limitations of already existing tools. It measures melanoma‐specific HRQoL and also takes into account the TRAE also of immunotherapy and targeted therapy. The EMQoLI is reliable and validated in German and it measures HRQoL in melanoma patients at all four tumor stages. It is a short and easy‐to‐use questionnaire that helps healthcare professionals to identify patients who may need further psychosocial support. Using the EMQoLI enables recognition of not only whether psychosocial support is needed, but also whether patients are potentially affected in the areas *Symptoms*, *Resources*, or *Burden*. Consequently, suitable interventions and referrals can be provided more efficiently. The EMQoLI therefore makes an important contribution to the psycho‐oncological care of people with melanoma.

## CONFLICT OF INTEREST STATEMENT

The authors declare the following financial interests/personal relationships which may be considered as potential competing interests:

**GL** reports advisory role or has received honoraria/travel costs from: Novartis, Pierre Fabre and Sunpharma.

**LZ** served as consultant and has received honoraria from BMS, MSD, Novartis, Pierre Fabre, Regeneron, and Sunpharma and travel support from MSD, BMS, Pierre Fabre, Regeneron, Sunpharma and Novartis, outside the submitted work.

**DS** reports grants or contracts from BMS, Amgen, MSD and Novartis, all to the institution. Consulting fees from BMS, Novartis, MSD, Moderna, BioNTech, Regeneron, Pierre Fabre, Pfizer, Boehringer Ingelheim, Replimune, Sunpharma, Philogen, Skyline Dx, Daiichi Sanyo, Ipsen, IoVance, IOBioTech, Immunocore, BioAlta, Immatics, Seagen and Merck‐Serono. Payment or honoraria for lectures, presentations, speakers bureaus, manuscript writing or educational events from BMS, Novartis, MSD, Pierre Fabre, Regeneron, Sunpharma and Regeneron. Support for attending meetings and/or travel from Pierre Fabre. Participation on a Data Safety Monitoring Board or Advisory Board from BMS, Novartis, MSD, Moderna, BioNTech, Regeneron, Pierre Fabre, Replimune, Philogen, Skyline Dx, Daiichi Sanyo, IMCheck, Ipsen, IoVance, IOBioTech, Immunocore, BioAlta and Immatics. Leadership or fiduciary role in other board, society, committee or advocacy group; paid or unpaid: Dermatologic Cooperative Group (DeCOG, unpaid), EuMelaREg (unpaid), NVKH (unpaid) and Deutsche Hautkrebsstiftung/Hiege‐Stiftung (unpaid).

**EMS** reports Sponsoring and Ad‐Board Activity for Novartis Pharma GmbH.

**EL** reports advisory role or has received honoraria/travel costs from Bristol‐Myers Squibb, Merck Sharp & Dohme, Kyowa Kirin, Novartis, Pierre Fabre, Regeneron, Sanofi, Sunpharma and Takeda.

**MT** reports Sponsoring and Ad‐Board Activity for Novartis Pharma GmbH.

If there are other authors, they declare that they have no known competing financial interests or personal relationships that could have appeared to influence the work reported in this paper.

## Supporting information



Supplementary information
