# Supplementary material for: Development and validation of the Essen Melanoma Quality of Life Inventory (EMQoLI)
Source: J Dtsch Dermatol Ges. 2026 Apr 20;24(9):1241–9. doi: 10.1111/ddg.15998 (PMC13551256; doi:10.1111/ddg.15998)
Supplement: Supplementary file 1 [file DDG-24-1241-s001.docx]

Supplemental online material

to

Development and Validation of the Essen Melanoma Quality of Life Inventory (EMQoLI)

Musche V., Schadendorf T., Schweda A., Lodde G., Neumann J., Zimmer L., Hüning S., Schadendorf D., Skoda E.-M., Livingstone E. & Teufel M.

**­­Supplemental results**

**Exploratory factorial analyses and item selection**

In the manuscript, we describe that an initial factorial analysis with a varimax rotation including all 33 items resulted in a rather suboptimal solution with cross-loadings across a number of items, as well as low communalities. Supplementary table S1 (see below) summarizes the results of such a solution. Please note that due to their high theoretical relevance and their centrality to quality of life, we decided to not exclude items 2 and 3 (“i have swellings in the area of the postoperative scar of the melanoma and / or the metastases” and “I suffer from tiredness“). Based on this, we subsequently excluded 8 items. Results of the exploratory factorial analysis after exclusion of the 8 items are presented in supplementary table S2. Here, we also included item communalities, as well as item-scale and item-total (Spearman-) correlations, indicating satisfying values.

**Reliability analyses.**

Reliability indices also indicate a satisfying level of reliability, with MacDonald’s Ωtotal - which was computed based on the same factorial structure as above. Here, we find values of 0.93, and a Cronbach’s α of 0.90. All three subscales show modest to moderate correlations with other subscales (Burden x Symptoms rho = .45, *p* < .001; Burden x Resources rho = 0.26, *p* < .001; Resources x Symptoms rho = 0.22, *p* < .001).

**Association of the EMQoLI subscales and sociodemographic data.**

In addition to testing for differences in EMQOLI scores for different stages of skin cancer, we also checked for patterns in sociodemographic variables. Here, further associations with education, family status, and employment status are reported. A Kruskal-Wallis-test that assesses the difference in the EMQoLI-scores across different levels of education indicates the existence of group differences in the EMQoLI total score (χ(4) = 11.158, *p* = .025), yet, subsequent Dunn’s tests only show increased levels of HRQoL burden in people with college degrees vs. people with high school degrees at an uncorrected level (i.e. without Holm-correction, *p* = .022). No effects could be found for family status (all *p* > .05). Regarding the participants’ employment status, people who indicate to still work part-time show higher EMQoLI total scores than retirees (χ ^2^(4) = 28.418, *p* < .001; Holm-corrected Dunn’s test *Z* = -3.362, *p* = .028; Holm-corrected). People in full-time employment and retired people exhibit less HRQoL burden than people on sick leave (*Z* = 3.309, *p* = .025; *Z* = 4.268, *p* < .001; Holm-corrected). Such patterns in the total score appear to result from differences in the subscales of *Symptoms* and *Burden*, with people on sick leave generally exhibiting more burden. Mann-Whitney-U tests show higher overall burden for each of the EMQoLI scales for people with diagnosed mental health conditions (all *p* < .001).

**Analysis of pilot data**

Prior to initiating full-scale data collection, we conducted a pilot study with a separate sample of 59 participants to obtain initial evidence for the reliability and validity of the EMQoLI. This pilot sample is independent and not included in the main analyses presented in this manuscript.

We conducted an initial inspection of item distributions and evaluated inter-item correlations as well as associations with external criteria to assess preliminary criterion validity. The majority of items demonstrated sufficient variance (see Supplemental Figure S1 and Supplemental Table S3), although several symptom-specific items exhibited lower variability, just as in the final validation sample. Given their relevance to melanoma treatments, these items were retained in the instrument. We further examined the overall pattern of inter-item correlations, which was predominantly positive (see Supplemental Figure S2 for Spearman correlations), and observed satisfactory internal consistency (Cronbach’s α = .90). Due to the limited sample size, we did not perform factorial analyses. To obtain a preliminary indication of criterion validity, we assessed correlations between the total EMQoLI score – i.e. here, the sum score of all items - and scores from the established instruments, i.e. the measures of impulsivity, the EORTC QLQ-C30, self-efficacy, depression, and anxiety (see Supplemental Figure S3). The results of this pilot study provided sufficient confidence to proceed with the main data collection.

**Preliminary English version of the Essen Melanoma Quality of Life Inventory (EMQoLI) for overview purposes only**

1. Pain at the surgery site
2. Swelling at the surgery site
3. Tiredness
4. Joint pain.
5. Loss of appetite.
6. Rash or itching.
7. Nausea.
8. Vomiting.
9. Diarrhea.
10. Fever.
11. Other adverse events.
12. Feeling supported by doctors
13. Fear of cancer returning/ condition worsening.
14. Hopeful about the future.
15. Satisfied with provided infomation by doctor‘s
16. Others did not understand my illness.
17. Fear of follow-up results.
18. Worried about outdoor activities in the sun
19. Supported by the healthcare system.
20. Worried about family’s melanoma risk.
21. Fear of dying.
22. Bothered by surgery scar.
23. Understood treatment options.
24. Pressure to check skin.
25. Supported by family and friends.

Supplemental Table S1

|  |  |  | Factor 1: Burden | Factor 2: Symptom Scale | Factor 3: Resources | Communality | Excluded |
| --- | --- | --- | --- | --- | --- | --- | --- |
| 1 | Pain at the surgery site |  | 0.53 | 0.10 | 0.07 | 0.295 |  |
| 2 | Swelling at the surgery site |  | 0.46 | 0.12 | 0.08 | 0.235 |  |
| 3 | Tiredness |  | 0.41 | 0.58 | 0.15 | 0.526 |  |
| 4 | Joint pain |  | 0.24 | 0.54 | 0.23 | 0.398 |  |
| 5 | Loss of appetite |  | 0.12 | 0.67 | -0.04 | 0.464 |  |
| 6 | Rash or itching |  | 0.27 | 0.45 | 0.03 | 0.280 |  |
| 7 | Nausea |  | 0.16 | 0.84 | 0.03 | 0.736 |  |
| 8 | Vomiting |  | 0.01 | 0.90 | -0.03 | 0.806 |  |
| 9 | Diarrhea |  | 0.16 | 0.70 | 0.08 | 0.524 |  |
| 10 | Fever |  | 0.08 | 0.77 | -0.04 | 0.594 |  |
| 11 | Other adverse events |  | 0.21 | 0.67 | 0.02 | 0.496 |  |
| 12 | Worried persistence adverse events |  | 0.41 | 0.50 | 0.09 | 0.422 | x |
| 13 | Feeling supported by doctors |  | -0.01 | 0.01 | 0.72 | 0.521 |  |
| 14 | Feeling depressed due to melanoma |  | 0.65 | 0.44 | 0.14 | 0.644 | x |
| 15 | Fear of cancer returning/ condition worsening |  | 0.82 | 0.23 | 0.01 | 0.724 |  |
| 16 | Restrictions due to melanoma |  | 0.56 | 0.53 | 0.17 | 0.623 | x |
| 17 | Hopeful about the future |  | 0.24 | 0.18 | 0.48 | 0.325 |  |
| 18 | Satisfied with provided information by the physicians |  | 0.13 | 0.09 | 0.80 | 0.669 |  |
| 19 | Others did not understand my illness |  | 0.63 | 0.19 | 0.32 | 0.538 |  |
| 20 | Fear of the follow-up examinations |  | 0.76 | 0.23 | 0.01 | 0.635 |  |
| 21 | Worried about outdoor activities in the sun |  | 0.59 | 0.19 | 0.05 | 0.391 |  |
| 22 | Guilty because not able to fulfill responsibilities |  | 0.58 | 0.46 | 0.24 | 0.604 | x |
| 23 | Supported by the healthcare system. |  | 0.03 | -0.01 | 0.54 | 0.294 |  |
| 24 | Worried about relatives melanoma risk |  | 0.55 | 0.13 | 0.02 | 0.317 |  |
| 25 | Uncomfortable being intimate |  | 0.44 | 0.46 | 0.24 | 0.460 | x |
| 26 | Regretting previous sun exposure |  | 0.45 | 0.07 | -0.05 | 0.208 | x |
| 27 | Fear of dying |  | 0.69 | 0.26 | 0.04 | 0.543 |  |
| 28 | Worried that coping with daily demands not possible |  | 0.55 | 0.56 | 0.14 | 0.639 | x |
| 29 | Bothered by surgery scar |  | 0.60 | 0.00 | 0.16 | 0.386 |  |
| 30 | Understood the treatment options |  | 0.02 | 0.07 | 0.70 | 0.501 |  |
| 31 | Pressure to check skin |  | 0.62 | 0.06 | 0.06 | 0.391 |  |
| 32 | Acceptance of illness |  | 0.05 | -0.04 | 0.47 | 0.223 | x |
| 33 | Supported by family and friends |  | 0.10 | 0.10 | 0.69 | 0.497 |  |

Factorial solution using an exploratory factor analysis with three factors, allowing for a varimax-rotation, on all 33 initial items. The shaded areas represent dominant loadings above 0.4, as well as communalities below the cutoff of 0.25.

Supplemental Table S2

|  |  |  | Factor 1: Burden |  | Factor 2: Symptom Scale |  | Factor 3: Resources |  | Communality | Item-scale correlation | Item-total correlation |
| --- | --- | --- | --- | --- | --- | --- | --- | --- | --- | --- | --- |
| 1 | Pain at the surgery site |  | 0.57 |  | 0.11 |  | 0.07 |  | 0.344 | 0.527 | 0.441 |
| 2 | Swelling at the surgery site |  | 0.49 |  | 0.12 |  | 0.06 |  | 0.263 | 0.459 | 0.392 |
| 3 | Tiredness |  | 0.40 |  | 0.56 |  | 0.17 |  | 0.499 | 0.801 | 0.623 |
| 4 | Joint pain |  | 0.23 |  | 0.52 |  | 0.25 |  | 0.387 | 0.709 | 0.516 |
| 5 | Loss of appetite |  | 0.13 |  | 0.68 |  | -0.02 |  | 0.484 | 0.554 | 0.351 |
| 6 | Rash or itching |  | 0.28 |  | 0.45 |  | 0.04 |  | 0.281 | 0.559 | 0.413 |
| 7 | Nausea |  | 0.17 |  | 0.86 |  | 0.06 |  | 0.766 | 0.562 | 0.429 |
| 8 | Vomiting |  | 0.01 |  | 0.90 |  | 0.00 |  | 0.808 | 0.334 | 0.257 |
| 9 | Diarrhea |  | 0.19 |  | 0.73 |  | 0.11 |  | 0.583 | 0.498 | 0.376 |
| 10 | Fever |  | 0.08 |  | 0.79 |  | -0.02 |  | 0.637 | 0.325 | 0.243 |
| 11 | Other adverse events |  | 0.21 |  | 0.62 |  | 0.03 |  | 0.435 | 0.578 | 0.385 |
| 12 | Feeling supported by doctors |  | 0.00 |  | -0.01 |  | 0.74 |  | 0.522 | 0.724 | 0.353 |
| 13 | Fear of cancer returning/ condition worsening |  | 0.78 |  | 0.22 |  | 0.01 |  | 0.657 | 0.775 | 0.655 |
| 14 | Hopeful about the future |  | 0.23 |  | 0.15 |  | 0.46 |  | 0.286 | 0.593 | 0.498 |
| 15 | Satisfied with provided information by the physicians |  | 0.13 |  | 0.06 |  | 0.86 |  | 0.757 | 0.750 | 0.471 |
| 16 | Others did not understand my illness |  | 0.64 |  | 0.17 |  | 0.32 |  | 0.540 | 0.603 | 0.612 |
| 17 | Fear of the follow-up examinations |  | 0.78 |  | 0.23 |  | 0.02 |  | 0.669 | 0.787 | 0.680 |
| 18 | Worried about outdoor activities in the sun |  | 0.59 |  | 0.20 |  | 0.06 |  | 0.390 | 0.651 | 0.555 |
| 19 | Supported by the healthcare system |  | 0.04 |  | -0.03 |  | 0.55 |  | 0.304 | 0.633 | 0.311 |
| 20 | Worried about relatives melanoma risk |  | 0.54 |  | 0.12 |  | 0.02 |  | 0.303 | 0.588 | 0.455 |
| 21 | Fear of dying |  | 0.66 |  | 0.25 |  | 0.07 |  | 0.504 | 0.728 | 0.626 |
| 22 | Bothered by surgery scar |  | 0.61 |  | -0.01 |  | 0.14 |  | 0.397 | 0.524 | 0.427 |
| 23 | Understood the treatment options |  | 0.03 |  | 0.05 |  | 0.67 |  | 0.456 | 0.678 | 0.367 |
| 24 | Pressure to check skin |  | 0.63 |  | 0.08 |  | 0.06 |  | 0.411 | 0.643 | 0.513 |
| 25 | Supported by family and friends |  | 0.11 |  | 0.09 |  | 0.67 |  | 0.464 | 0.597 | 0.389 |
|  |  |  |  |  |  |  |  |  |  |  |  |

Final factorial solution using an exploratory factor analysis with three factors on remaining 25 items after exclusion of items in factorial analysis showed in supplementary table S1. Again, the shaded areas represent dominant loadings above 0.4. Communalities, as well as item-scale and item-total correlations show satisfying values.

Supplemental Table S3

|  |  | M | SD |  | Median | 25. Percentile | 75. Percentile | Skew | Kurtosis |
| --- | --- | --- | --- | --- | --- | --- | --- | --- | --- |
| 1 | Pain at the surgery site | 0.75 | 0.92 |  | 0 | 0 | 1 | 0.95 | -0.19 |
| 2 | Swelling at the surgery site | 0.61 | 1.03 |  | 0 | 0 | 1 | 1.92 | 3.17 |
| 3 | Tiredness | 1.25 | 1.03 |  | 1 | 1 | 2 | 0.65 | -0.26 |
| 4 | Joint pain | 1.05 | 1.33 |  | 0 | 0 | 2 | 0.99 | -0.23 |
| 5 | Loss of appetite | 0.24 | 0.60 |  | 0 | 0 | 0 | 2.39 | 4.33 |
| 6 | Rash or itching | 0.81 | 1.21 |  | 0 | 0 | 1 | 1.52 | 1.35 |
| 7 | Nausea | 0.24 | 0.68 |  | 0 | 0 | 0 | 3.79 | 16.90 |
| 8 | Vomiting | 0.14 | 0.60 |  | 0 | 0 | 0 | 5.39 | 31.57 |
| 9 | Diarrhea | 0.47 | 1.02 |  | 0 | 0 | 0 | 2.42 | 5.45 |
| 10 | Fever | 0.12 | 0.49 |  | 0 | 0 | 0 | 4.71 | 23.32 |
| 11 | Other adverse events | 0.34 | 0.84 |  | 0 | 0 | 0 | 2.85 | 8.08 |
| 12 | Worried persistence adverse events | 0.85 | 1.16 |  | 0 | 0 | 1 | 1.35 | 1.02 |
| 13 | Feeling supported by doctors | 0.88 | 1.20 |  | 0 | 0 | 1 | 1.28 | 0.59 |
| 14 | Feeling depressed due to melanoma | 1.03 | 1.11 |  | 1 | 0 | 2 | 1.17 | 1.15 |
| 15 | Fear of cancer returning/ condition worsening | 1.92 | 1.26 |  | 2 | 1 | 3 | 0.32 | -1.05 |
| 16 | Restrictions due to melanoma | 0.97 | 1.07 |  | 1 | 0 | 1 | 1.22 | 0.99 |
| 17 | Hopeful about the future | 1.71 | 1.47 |  | 2 | 0 | 3 | 0.32 | -1.26 |
| 18 | Satisfied with provided information by the physicians | 1.10 | 1.39 |  | 1 | 0 | 2 | 1.10 | -0.10 |
| 19 | Others did not understand my illness | 0.97 | 1.31 |  | 0 | 0 | 2 | 1.01 | -0.40 |
| 20 | Fear of the follow-up examinations | 1.71 | 1.35 |  | 2 | 1 | 3 | 0.25 | -1.17 |
| 21 | Worried about outdoor activities in the sun | 1.54 | 1.36 |  | 1 | 0 | 2 | 0.42 | -0.95 |
| 22 | Guilty because not able to fulfill responsibilities | 0.71 | 1.16 |  | 0 | 0 | 1 | 1.55 | 1.23 |
| 23 | Supported by the healthcare system. | 1.44 | 1.28 |  | 1 | 0.5 | 2 | 0.70 | -0.53 |
| 24 | Worried about relatives melanoma risk | 1.61 | 1.30 |  | 1 | 1 | 3 | 0.33 | -1.10 |
| 25 | Uncomfortable being intimate | 0.59 | 1.10 |  | 0 | 0 | 1 | 2.00 | 3.26 |
| 26 | Regretting previous sun exposure | 1.75 | 1.45 |  | 2 | 0 | 3 | 0.14 | -1.40 |
| 27 | Fear of dying | 1.66 | 1.38 |  | 1 | 1 | 3 | 0.48 | -1.07 |
| 28 | Worried that coping with daily demands not possible | 1.08 | 1.25 |  | 1 | 0 | 2 | 1.04 | 0.04 |
| 29 | Bothered by surgery scar | 0.80 | 1.23 |  | 0 | 0 | 1 | 1.44 | 0.85 |
| 30 | Understood the treatment options | 0.88 | 1.20 |  | 0 | 0 | 2 | 1.22 | 0.46 |
| 31 | Pressure to check skin | 1.08 | 1.16 |  | 1 | 0 | 2 | 0.92 | 0.09 |
| 32 | Acceptance of illness | 1.22 | 1.29 |  | 1 | 0 | 2 | 0.58 | -0.92 |
| 33 | Supported by family and friends | 0.97 | 1.39 |  | 0 | 0 | 2 | 1.18 | 0.00 |

Descriptive statistics of the items of the 59 participants that were collected separately in the pilot study prior to the actual recruitment (see also supplemental figure S1)

Supplemental figure S1

Distributions of EMQoLI-items in the pilot data

Supplemental figure S2

Spearman correlations between all EMQoLI items in pilot dataset (n = 59), showing an overall positive correlational pattern.

Supplemental figure S3

Spearman correlation coefficients between all scales used in the pilot study with n = 59. Note that the correlations between the sum of all EMQoLI items and the other measurements roughly represent the associations found in the final validation study as presented in the main manuscript, with comparably low correlations with the BIS, significantly positive associations with the EORTC instrument, as well as anxiety and depression, and a negative relationship with general self-efficacy.

|  |  |  |
| --- | --- | --- |

Essen Melanoma Quality of Life Inventory (EMQoLI)

Im Folgenden finden Sie eine Reihe von Aussagen zu verschiedenen Lebensbereichen, die durch Ihre Melanomerkrankung betroffen sein können. Bitte lesen Sie sich jede Aussage in Ruhe durch und beurteilen Sie, wie sehr (0 = gar nicht bis 4 = sehr stark) diese in den letzten 14 Tagen einschließlich heute auf Sie zutraf. Bitte bearbeiten Sie die Aussagen vollständig und lassen Sie keine aus.

|  | **Gar nicht** | **Ein wenig** | **Teil-weise** | **Etwas mehr** | **Sehr stark** |
| --- | --- | --- | --- | --- | --- |
| 1. Ich hatte Schmerzen im Bereich der Operationsnarbe des Melanoms und/oder der Metastasen. | ☐ 0 | ☐ 1 | ☐ 2 | ☐ 3 | ☐ 4 |
| 2. Ich hatte Schwellungen im Bereich der Operationsnarbe des Melanoms und/oder der Metastasen. | ☐ 0 | ☐ 1 | ☐ 2 | ☐ 3 | ☐ 4 |
| 3. Ich litt unter Müdigkeit. | ☐ 0 | ☐ 1 | ☐ 2 | ☐ 3 | ☐ 4 |
| 4. Ich litt unter Gelenkschmerzen. | ☐ 0 | ☐ 1 | ☐ 2 | ☐ 3 | ☐ 4 |
| 5. Ich litt unter Appetitlosigkeit. | ☐ 0 | ☐ 1 | ☐ 2 | ☐ 3 | ☐ 4 |
| 6. Ich litt unter Hautausschlag und/oder Juckreiz. | ☐ 0 | ☐ 1 | ☐ 2 | ☐ 3 | ☐ 4 |
| 7. Ich litt unter Übelkeit. | ☐ 0 | ☐ 1 | ☐ 2 | ☐ 3 | ☐ 4 |
| 8. Ich litt unter Erbrechen. | ☐ 0 | ☐ 1 | ☐ 2 | ☐ 3 | ☐ 4 |
| 9. Ich litt unter Durchfall. | ☐ 0 | ☐ 1 | ☐ 2 | ☐ 3 | ☐ 4 |
| 10. Ich litt unter Fieber. | ☐ 0 | ☐ 1 | ☐ 2 | ☐ 3 | ☐ 4 |
| 11. Ich litt unter weiteren Nebenwirkungen der Behandlung.   Weitere Nebenwirkungen: ____________________________________________  ____________________________________________ | ☐ 0 | ☐ 1 | ☐ 2 | ☐ 3 | ☐ 4 |
|  | **Gar nicht** | **Ein wenig** | **Teil-weise** | **Etwas mehr** | **Sehr stark** |
| 12. Ich fühlte mich durch die behandelnden Ärzte unterstützt. | ☐ 0 | ☐ 1 | ☐ 2 | ☐ 3 | ☐ 4 |
| 13. Ich hatte Angst vor einem Wiederauftreten der Erkrankung oder der Verschlechterung meines Zustandes. | ☐ 0 | ☐ 1 | ☐ 2 | ☐ 3 | ☐ 4 |
| 14. Ich habe hoffnungsvoll in die Zukunft geblickt. | ☐ 0 | ☐ 1 | ☐ 2 | ☐ 3 | ☐ 4 |
| 15. Ich war mit der Aufklärung durch die behandelnden Ärzte zufrieden. | ☐ 0 | ☐ 1 | ☐ 2 | ☐ 3 | ☐ 4 |
| 16. Es hat mich belastet, dass Andere (z.B. Arbeitskollegen, Freunde) die Schwere der Melanomerkrankung nicht erkennen. | ☐ 0 | ☐ 1 | ☐ 2 | ☐ 3 | ☐ 4 |
| 17. Ich hatte Angst vor dem Ergebnis der Folgeuntersuchungen. | ☐ 0 | ☐ 1 | ☐ 2 | ☐ 3 | ☐ 4 |
| 18. Ich hatte Bedenken, (Freizeit-)Aktivitäten in der Sonne nachzugehen (z.B. Spazierengehen, Sport, Urlaubsreisen). | ☐ 0 | ☐ 1 | ☐ 2 | ☐ 3 | ☐ 4 |
| 19. Ich fühlte mich vom Gesundheitssystem unterstützt (z.B. psychologische Unterstützung, Sozialdienst, Rehabilitationsangebot). | ☐ 0 | ☐ 1 | ☐ 2 | ☐ 3 | ☐ 4 |
| 20. Ich machte mir Sorgen um ein erhöhtes Melanomrisiko meiner Angehörigen. | ☐ 0 | ☐ 1 | ☐ 2 | ☐ 3 | ☐ 4 |
| 21. Ich hatte Angst, aufgrund meiner Erkrankung zu sterben. | ☐ 0 | ☐ 1 | ☐ 2 | ☐ 3 | ☐ 4 |
| 22. Die Narbe der Operation belastete mich. | ☐ 0 | ☐ 1 | ☐ 2 | ☐ 3 | ☐ 4 |
| 23. Ich hatte das Gefühl, die Behandlungsmöglichkeiten ausreichend gut verstanden zu haben. | ☐ 0 | ☐ 1 | ☐ 2 | ☐ 3 | ☐ 4 |
| 24. Ich empfand Druck, meine Haut ständig nach neuen Veränderungen (z.B. Rötungen, Leberflecke) untersuchen zu müssen. | ☐ 0 | ☐ 1 | ☐ 2 | ☐ 3 | ☐ 4 |
| 25. Ich fühlte mich durch mein privates Umfeld (z.B. Familie, Freunde) unterstützt. | ☐ 0 | ☐ 1 | ☐ 2 | ☐ 3 | ☐ 4 |

|  |  |  |
| --- | --- | --- |

Essen Melanoma Quality of Life Inventory (EMQoLI)

Für Behandler:innen – nicht für Patient:innen

Auswertung:

Höhere EMQoLI-Werte weisen auf eine stärkere Beeinträchtigung der gesundheitsbezogenen Lebensqualität hin.

Invertierte Fragebogen-Items: 12, 14, 15, 19, 23, 25

Skalen:

Symptome: Fragebogen-Items 3 – 11

Belastung: Fragebogen-Items 1, 2, 13, 16, 17, 18, 20, 21, 22, 24

Ressourcen: Fragebogen-Items 12, 14, 15, 19, 23, 25 (alle invertiert)

­­
